# Measuring the mediating role of quality education for ensuring employability skills: An analysis of higher education student perception in Bangladesh

**DOI:** 10.1371/journal.pone.0310815

**Published:** 2024-10-07

**Authors:** Khandakar Kamrul Hasan, Shadia Sharmin, A. T. M. Fahimul Islam, Hissan Khandakar, Abdul Hasib Siddique, Ariful Hoque Shuhan, Mobashwer A. Khandaker

**Affiliations:** 1 Department of Educational Leadership, Policy and Technology Studies, The University of Alabama, Tuscaloosa, Alabama, United States of America; 2 Department of Business Administration, University of Scholars, Banani, Dhaka, Bangladesh; 3 Faculty of Business Studies, Bangladesh University of Professionals (BUP), Mirpur Cantonment, Dhaka, Bangladesh; 4 Cambridge Assessment International Education A Level, British Council, Nilkhet, Dhaka, Bangladesh; 5 Department of Electrical and Electronics Engineering, University of Scholars, Banani, Dhaka, Bangladesh; 6 Office of the Registrar, University of Scholars, Banani, Dhaka, Bangladesh; Universidade do Sul de Santa Catarina, BRAZIL

## Abstract

The present study employs a quantitative approach to measure the student perception of the direct and mediating effect of quality education for ensuring employability skills in higher education students of Bangladesh. The study was conducted on 154 undergraduate and graduate students at a Private University in Bangladesh, through a cross-sectional survey using a structured data questionnaire. The multilevel measurement and structural model, which was based on the constructs of quality education, employability skills of students, course structure, institutional policy, and physical aspects, was analysed using Partial Least Squares modelling with SmartPLS 4. The aim was to identify the employability skills that are present among higher education students in Bangladesh through student perception and explore the mediating role of quality education in shaping these skills. The findings suggest a significant gap between the skills taught in private universities, and the industry requirements of Bangladesh, which highlights the urgency for administrators and policy-makers to act fast. physical aspects have a positive influence on quality education and employability skills, but course structure and policy show less direct impact. Moreover, quality education is a crucial mediator for only the factors that match a direct effect. This proves that higher education students in Bangladesh may not have acquired the technical knowledge required by the industry. However, the present study was conducted on undergraduate and graduate students at a single private university, thus acknowledging the need to diversify the population sample in future studies for enhanced generalizability. The implications of these results extend to educational policymakers, institutions, and stakeholders, therefore emphasizing the need for curriculum enhancement, industry-academia collaboration, and policy reforms.

## Introduction

There are currently 54 Public Universities and 112 Private Universities in Bangladesh [1, 2]. Even though Public Universities are considered some of the most prestigious institutions in the country, only 202,421 seats are available each year, and a further 266,625 are available each year from Private Universities [3]. The rest of the prospective students attend National Universities, with around 1,268,322 seats yearly [3]. Around 1,034,328 Students who passed the Higher Secondary Certificate Examinations in 2022 must be accommodated in these seats [4]. Despite the proliferation of private universities, there is a growing concern that the education they offer may need to be aligned with the needs of employers, leading to a skills gap among graduates. This misalignment poses a significant challenge for the students, educational institutions, and policymakers responsible for shaping the country’s future workforce [5].

The exploration of employability skills among higher education students in Bangladesh has only recently garnered scholarly interest [6–8]. The National Education Policy 2010 (NEP-2010) stressed the importance of aligning education with labor market demands [9]. However, the effectiveness of this policy remains questionable. As noted by [10], several stakeholders have struggled to maintain educational standards as per NEP-2010. In addition [8], observed that the quality of education significantly influences students’ employability skills, stating that the "Bangladeshi education system is old-fashioned with no accommodation for skill dynamics, technology, and the labour market." This is juxtaposed with findings from other contexts, such as [11], which identified student over-confidence as a major barrier to employability skills, underscoring the disparity within Bangladeshi HEIs. Despite these insights, the current study’s scope is limited, with data derived from a single school, thus not fully achieving the goal of identifying the employability skills across the higher education spectrum in Bangladesh.

Nevertheless, the inquiry persists: Do the graduates of these private universities possess sufficient readiness for the labour market? This research paper explores this critical issue through a quantitative study utilising a structured questionnaire and cross-sectional data sampling to collect data about students’ perceptions of quality education in their institutions. A comprehensive literature review showcases how different institutions provide quality education to their students to ensure employability skills. Moreover, the direct relationship between Quality Education and Employability Skills of Students will also be discussed. The primary research problem this study aims to address is the factors determining university students’ Quality Education and its influence.

The study is necessary due to its quantitative insights into the impact of quality education on higher education (HE) students. This study aims to contribute to ongoing efforts to improve the quality of education and develop employability skills among individuals. This study’s findings can be a substantial resource for educational institutions, policymakers, and other relevant stakeholders actively changing Bangladesh’s education trajectory.

### Research questions

The objectives of the study are as follows:

- The purpose of this study is to examine the factors determining university students’ Quality Education (QE) by investigating the relationship between Course Structure (CS), Institutional Policy (Pol), and Physical Aspects (PA) to ensure Employability Skills of Students (ES) in higher education.
- To explore the influence of Quality Education (QE) as a mediator in the relationship between exogenous and endogenous factors.

## Literature review

In alignment with the principles of Student-Centered Learning (SCL), this approach aims to assess the educational experience from the students’ perspectives comprehensively. SCL is a pedagogical approach that places students at the core of the learning process, emphasising active participation, collaboration, critical thinking, and self-directed learning [12]. Since SCL is a complex learning process, students must be thoroughly supported in the motivational, cognitive, and social aspects. Interestingly, this is precisely the approach that must be taken by institutions to provide quality education to ensure employability skills by encouraging both soft and hard skills, as well as collaboration. The constructs of Quality Education, Physical Aspect, Course Structure and Institutional Policy have been developed to assess the quality of education that is being provided by the private university sector of Bangladesh, which itself has been adapted from the SERVQUAL instrument put forward by [13]. The research variables used for this paper have been identified based on the adaptation of [14], shown in Table 1.

**Table 1:** Identification of research variables.

Constructs	Meaning	Level of Measurement	Role	Sample Indicators	Source
**Employability Skills of Students**	This construct encompasses skills like collaboration, respectful engagement, constructive feedback, adept technology use, goal attainment, and self-directed multitasking.	Individual (Students)	Dependent	Number of collaborative projects participated in Use of technology in coursework.	[11]
**Quality Education**	A measure of the institution’s professionalism and skill development offerings, essential for students’ careers. It involves active student participation and rigorous assessment across diverse courses.	Institutional	Mediating	Variety of courses offered. Student engagement in institutional activities.	[15]
**Course Structure**	Courses designed to meet industry requirements, fostering communication skills and teamwork among students.	Institutional	Independent	Alignment with industry needs. Team-based project components	[16]
**Institutional Policy**	The degree to which the institution provides financial aid and student assistantship opportunities.	Institutional	Independent	Percentage of students receiving financial aid. Number of assistantship positions available.	[17]
**Physical Aspects**	The aesthetic appeal of the campus, availability of facilities, support services, and overall student safety on campus.	Institutional	Independent	Campus safety reports. Availability of recreational facilities.	[18]

### Course structure (CS) and its relationship with quality education (QE)

Various aims and agendas are established at the international, national, and regional levels to guarantee education provision. A comprehensive education should possess the capacity to impart fundamental knowledge and competencies necessary for the advancement of individuals. It should aid in cultivating positive habits and values and fostering logical, critical, and autonomous thinking abilities. Furthermore, it should serve as a catalyst to inspire and unleash individuals’ potential, creativity, and curiosity, encouraging them to explore and uncover novel knowledge for the betterment of humanity and the global community [19]. Nonetheless, the learning problem remains pervasive in contemporary times, with over 250 million children of school age worldwide failing to acquire fundamental quantifiable competencies, such as reading and numeracy. Half of these children had received formal education for at least four years. The presence of substandard education that hinders the achievement of fundamental learning objectives is seen in many countries and areas, including Arab and African nations, as well as affluent OECD (Organization for Economic Co-operation and Development) countries [19]. Research conducted on an award-winning instructor in the United States has determined that aligning course structure with its intended goal is crucial. The study also emphasises the significance of a conceptual framework that centres on online course design, evaluation and assessment, and facilitation. Furthermore, the study suggests reviewing relevant literature in the context of this framework [20]. Thus, the following hypothesis is posited:

- **H1**: Student perception of course structure positively affects the employability skills of higher education students.

### Institutional policy (POL) and its relationship with quality education (QE)

According to [21], enhancing graduate employability and developing competencies globally require robust innovation and collaborative practices inside higher education institutions. Nevertheless, the investigation’s outcomes suggest a significant gap must be addressed in higher education institutions’ adoption of sustainable policies on this subject matter.

The National Education Policy 2010 (NEP-2010) in Bangladesh acknowledged aligning education with the labour market requirements. It underscored the significance of this alignment of imparting skills and competencies to enhance employability [9]. Nevertheless, the efficacy of the policy raises concerns, as highlighted by [10], due to the inability of several stakeholders to uphold educational standards under the NEP-2010. Consequently, numerous individuals with university qualifications need help securing employment opportunities. The employers assert that graduates lack the necessary skill sets.

The investigation of methodologies employed in quality assessments and the corresponding critiques holds significance for several stakeholders within higher education, encompassing policymakers tasked with reviewing assessment outcomes [17]. There is a growing demand to hold policymakers accountable among other stakeholders “for greater public transparency of assessment results” [22, 23]. The prevailing climatic conditions have consequently given rise to a commensurate requirement for increased and enhanced knowledge about evaluation methodologies and alternative options for assessing quality. Institutional investments in assessments encompass allocating faculty and administrative time and resources to develop, administer, and disseminate results. This continuous gathering of information about program experiences and effectiveness plays a crucial role in providing the requisite context for interpreting the findings of new studies and guiding policy changes at the institutional or program level [14].

A past researcher [17] opined that the most remarkable advancement in quality assessments has been achieved in "evaluating student experiences and outcomes because the measures offer more than a description of incoming students’ talents and abilities." Nevertheless, further research is required to explore other methods of evaluating the effectiveness of educational initiatives beyond relying just on student feedback. This is crucial for facilitating comparisons across different institutions, establishing a connection between present-day programs and their long-term consequences, and informing legislative efforts. Thus, the following hypothesis is posited:

- **H2**: Student perception of institutional policy positively affects the employability skills of higher education students.

### Physical aspects (PA) and its relationship with quality education (QE)

Research findings substantiate that the physical dimension holds greater significance in higher education. This is primarily due to the pivotal role played by the quality and quantity of resources and amenities, including workshops, laboratories, libraries, and computer and information systems, in facilitating the learning and teaching processes. Additionally, auxiliary facilities such as accommodations, sports centres, restaurants, and the overall environment contribute to the overall educational experience [13, 24]. In an optimal educational setting, it is crucial to possess sufficient infrastructure to facilitate effective teaching and learning [25]. Numerous studies have demonstrated that the absence and insufficiency of these amenities significantly impact the academic outcomes of both students and instructors inside tertiary educational establishments [26].

Previous research on primary and secondary educational institutions has consistently demonstrated the significance of the physical campus environment in facilitating effective teaching and learning. These studies have further substantiated the existence of strong positive associations between the quality of school amenities and students’ academic performance [27–29]. According to [30], past researchers had to adopt a post-occupancy evaluation (POE) approach to assess the quality of the HEI campus and services. A comprehensive POE technique employs only a handful of methods, such as facilities management (FM) performance against functional, financial, technical, and behavioural criteria, that are uniquely adapted to evaluating the performance of educational facilities [31, 32]. Thus, the following hypothesis is posited:

- **H3**: Student perception of physical aspects positively affects the employability skills of higher education students.

### Quality education (QE) and its relationship with employability skills of students (ES)

Education increases production and income, boosting wealth; however, identifying acceptable education indicators and settings is challenging for developing countries to establish a quality evaluation system, which is why several views and reasoning frameworks are needed to evaluate educational policies to encourage education in poor nations and worldwide [33].

Undoubtedly, quality education is essential for developing individuals and the nation. While there is a considerable amount of literature on the importance of employability skills and quality education [5, 8], there needs to be more research focusing on private universities in Bangladesh. Most existing studies either concentrate on public universities or provide a generalised overview without diving into the intricacies of the private education sector. In addition, there is a need for more scholarly investigations that utilise a theme analysis methodology to examine the experiences and perspectives of students concerning student-centred learning (SCL) as a strategy for improving educational quality and fostering the acquisition of employable skills [8]. Higher education is of the highest importance as it significantly influences the development of employability skills among graduates [5]. As the global economy intensifies in competitiveness, there is an unprecedented need for a highly proficient labour force [34]. Considering the increasing accessibility of information, the growing prevalence of competition, and the ubiquitous influence of technology, the significance of learning is heightened as a prerequisite for active engagement in the contemporary knowledge-based economy [35]. This phenomenon holds particularly true in developing nations such as Bangladesh, where there has been a notable expansion in the private education industry in recent years [8].

Private institutions have become increasingly favoured among students pursuing higher education due to their diverse selection of courses and areas of expertise. Nevertheless, the exponential growth of these educational establishments has prompted inquiries regarding the calibre of instruction they offer and its applicability inside the labour market [5]. Thus, the following hypothesis is posited:

- **H4**: Student perception of Quality education positively affects the employability skills of higher education students.

### The mediating role of quality education (QE)

An alarming 2.3 million graduates are jobless in Bangladesh’s higher Education and labour market dynamics. Moreover, 350,000 graduates enter the labour market yearly, yet 200,000 remain unemployed. This has raised the graduate unemployment rate from 32% (2015) to 47% in 2019 [36]. According to [37], the elevated level of graduate unemployment in Bangladesh can be ascribed to many factors. These factors encompass a diminished demand for graduates within the labour market, exacerbated by a surplus of graduates, insufficient professional competencies among graduates, inefficiencies in the educational system, and outdated curricula, among others [37]. The Centre for Policy Dialogue (CPD) reports that a concerning trend persists in which approximately 5 out of every ten graduates in Bangladesh find themselves unemployed [7]. In contrast, India and Pakistan face a comparatively lower rate, with 3 out of every five graduates remaining unemployed [38]. Understanding this disparity may help HEIs ensure employability skills for graduate students.

The conversation around HE has historically been centred around the employability discussion since the primary objective of HE institutions is to produce graduates who possess the necessary skills and qualifications for the workforce [39]. The employability of university graduates is a topic of significant interest among scholars, legislators, and curriculum developers worldwide, owing to its substantial significance over the last decade [40]. The Bologna Process, which rose to prominence due to significant concern regarding the elevated levels of unemployment among graduates in certain European nations, emphasises the evaluation of HEIs based on the employability of their graduates on par with the quality of education provided by these HEIs [41]. However, prior literature needs to adopt the Service Quality (SERVQUAL) Instrument, which was adapted from [13], as Quality Education (QE). Moreover, even though evidence suggests that QE may mediate in ensuring employability skills [39–41], prior literature needs to discuss its significance. Thus, the following hypotheses are posited:

- **H5**: Student perception of Quality education and the mediating relationship between physical aspects and employability skills of higher education students.
- **H6**: Student perception of Quality education and the mediating relationship between course structure and employability skills of higher education students.
- **H7**: Student perception of Quality education and the mediating relationship between institutional policy and employability skills of higher education students.

**Fig 1:**
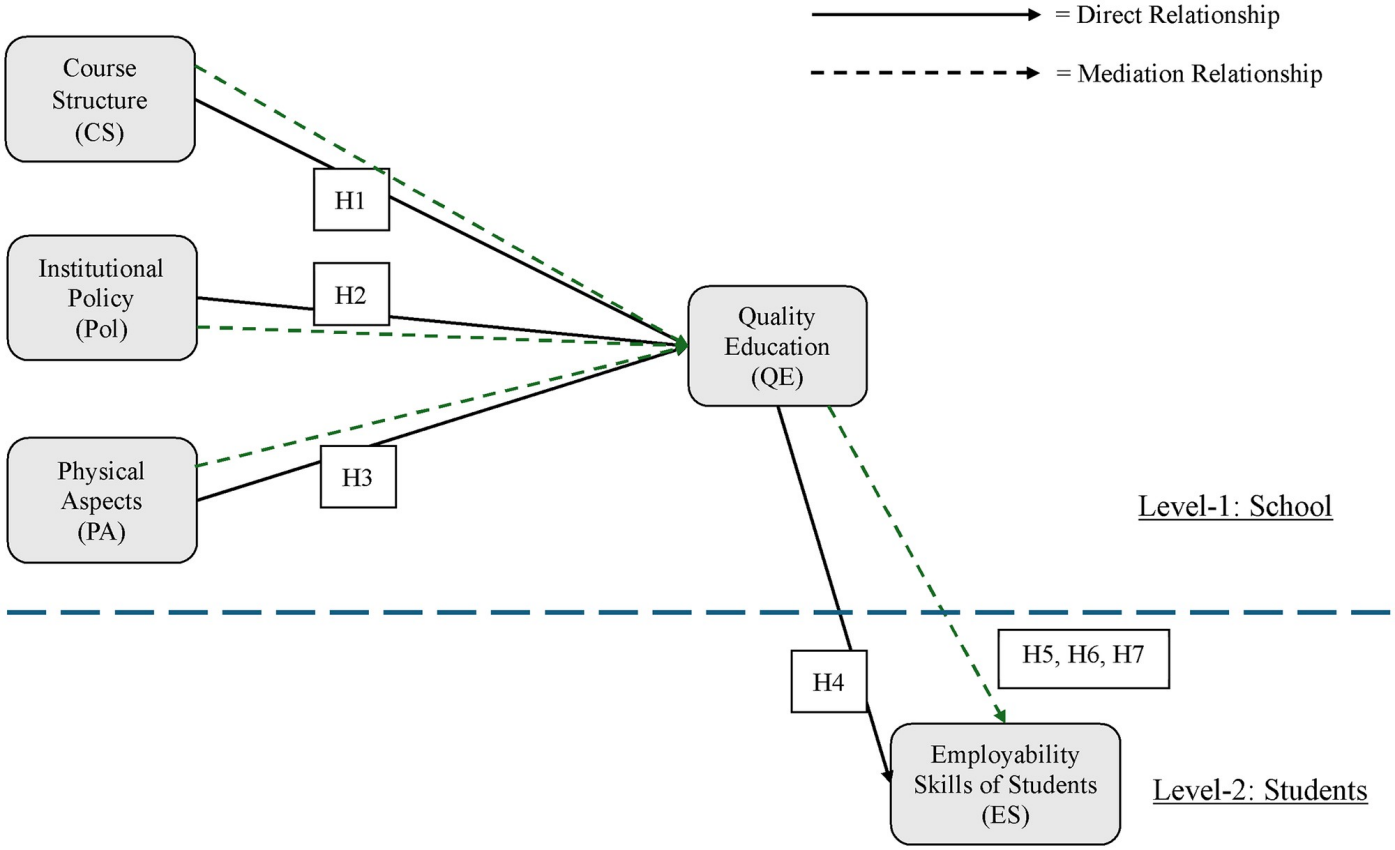
The mediating role of quality education for ensuring employability skills in higher education students.

Fig 1 illustrates the conceptual model for this investigation in accordance with the proposed hypotheses, the SCL Theory, the theoretical discussion, and the empirical findings of prior research.

## Methodology

### Ethics statement

The study has been approved by the Research Committee at the University of Scholars, Bangladesh, as the research is being conducted on human participants, i.e., students of a private university. Moreover, consent from the participants were taken through Google Forms, wherein participants were informed that every question is entirely voluntary, and that the survey does not collect any personally identifying information as responses will remain strictly anonymous. Submission of the Google Form was counted as participant consent.

### Sample and data collection

The study has been carried out at the University of Scholars, Dhaka. The respondents are students pursuing bachelor’s and master’s programs in the Department of Business Administration and have at least minimum employability experience in part-time or full-time jobs, respectively. The Google Form was disseminated and data from students was collected, starting from 1^st^ December 2023, until 31^st^ December 2023, flexibly over a span of 31 days. A total of 154 students responded. Among them, 10 data were discarded for spurious nature. The final data considered 144 participants for further analysis. The valid 144 cases comprise 108 males (75 per cent) and 36 females (25 per cent). According to [42], the prescribed range of 100–150 respondents for obtaining dependable results from structural equation models, the current study’s sample size is adequate.

A cross-sectional survey of University of Scholars students was undertaken using the non-probability purposive sampling method. A structured questionnaire was constructed to collect responses for this study. The constructs of employability skills of students (ES), quality education (QE), course structure (CS), institutional policy (POL), and physical aspects (PA) have a total of 31 question items. Respondents were required to respond with the statements provided using the seven-point Likert scale ranging from 1 (strongly disagree) to 7 (strongly agree).

Prior research [43] state that power analysis is frequently suggested in the PLS-SEM literature for determining the sample size for structural equation modelling. [44] suggest that to determine the minimal sample size, one should employ power analysis and consider the model constructs with the greatest number of significant predictors. According to [44], the rule of thumb established by [45] helps assess the statistical power of multiple regression models and determine the appropriate sample size. This rule considers the path model’s complexity, a minimum R2 value, and 80% statistical power. A minimum sample size of 103 was necessary in the PLS path model employed in this investigation to identify a minimum R2 value of 0.10 at a significance level of 5% and a suggested statistical power of 80%.

### Measures

From the literature, the measurement items for all constructs were adapted. The purpose of this study is to identify the relationship among Course Structure (CS), Institutional Policy (Pol), Physical Aspects (PA), Quality Education (QE), and Employability Skills of Students (ES), as well as the mediating role of Quality Education (QE), the unit of analysis was at the individual level. This study analysed all path linkages in the research model for hypothesis testing. The approach utilised in this research is confirmatory factor analysis (CFA), as the constructs incorporated in this model have been borrowed from previous investigations. PLS has the potential for two general applications, according to [46]: theory development or theory confirmation. PLS generates hypotheses in the latter scenario by investigating the interrelationships among variables.

Furthermore, PLS methods are frequently preferable when the phenomenon being investigated is relatively novel or evolving or when the theoretical model or measures are not well-defined [47]. Additionally [48], argue that PLS is more appropriate for developing theories rather than for verifying them. Thus, employing the CFA method in the present investigation is reasonable.

## Results

### Data analysis

The statistical method used to analyze the measurement and structural model was partial least squares (PLS) modeling with SmartPLS 4.0.9.6 [49]. This choice was made because PLS modeling does not necessitate the assumption of normality, which is often absent in survey research [50]. SPSS regression was deemed inadequate due to the complex model involving mediation, which couldn’t be tested simultaneously. Structural equation modeling was therefore implemented.

Prior research [43, 51, 52] outlined the following benefits of utilizing PLS-SEM:

- PLS-SEM can operate with a limited sample size.
- PLS-SEM evaluation of mediation is superior to regression analysis.

According to [48, 51], PLS-SEM is particularly well-suited for complex models where prediction is of greater importance than parameter estimation, and conditions such as sample size, independence, or normal distribution are not satisfied. Thus, it was appropriate to employ PLS-SEM in this investigation. The multilevel model was used for the analysis of data with a hierarchical or cluster structure and is popular in educational research [54]. This multilevel model is required because “Students” are generally considered a Level-2 analysis unit in educational research [55].

In adherence to the recommendations [53, 54], the concern of Common Method Bias was mitigated by conducting a comprehensive collinearity test since the data were obtained from a single source. By employing this approach, each variable is regressed against a standard variable; the absence of bias from a single data source is confirmed when the VIF falls below 3.3. As the analysis produced a VIF below 3.3, single-source bias did not appear to be a significant concern with these data.

A significant concern for the multilevel conceptual model was the potential for Omitted Variable Bias at the individual level [55], especially since the “Student” level had no other control variables. However, as justified by [56], it is impractical to collect all requisite data since the present study is strictly observational.

### Measurement model

Based on the recommendations put forth by [57], the constructed model underwent two-step testing. Before proceeding, the instrument’s validity and reliability were assessed on the measurement model per the protocols outlined by [51, 58]. Then, the developed hypothesis was evaluated by executing the structural model.

The loadings, average variance extracted (AVE), and composite reliability (CR) for the measurement model were evaluated. The CR should exceed 0.7, the loading values should exceed 0.5, and the AVE value should exceed 0.5. AVEs and CRs exceeded 0.5 and 0.7, respectively, as shown in Table 2.

**Table 2:** Measurement of construct validity* (Source: Authors).

Construct	Items	Loadings	CR	AVE
**Course Structure**	CS1	0.717	0.823	0.609
	CS2	0.783		
	CS3	0.836		
**Employability Skills of Students**	ES1	0.716	0.836	0.507
	ES2	0.797		
	ES3	0.64		
	ES5	0.654		
	ES7	0.743		
**Physical Aspects (PA)**	PA1	0.752	0.798	0.500
	PA2	0.659		
	PA4	0.776		
	PA5	0.629		
**Institutional Policy**	Pol1	0.691	0.808	0.585
	Pol2	0.783		
	Pol3	0.816		
**Quality Education**	QE1	0.742	0.857	0.500
	QE2	0.75		
	QE3	0.777		
	QE4	0.667		
	QE5	0.643		
	QE7	0.652		

* **Note**: Two items were dropped from employability skills of students (ES4, ES6), one item was dropped from physical aspects (PA3), and one item was dropped from quality education (QE6) because of their low loadings (below 0.5).

#### Convergent validity

The Composite Reliability (CR) values for Employability Skills of Students (ES), Quality Education (QE), Course Structure (CS), Institutional Policy (Pol), and Physical Aspects (PA) were 0.836, 0.857, 0.823, 0.891, 0.808 and 0.798, respectively indicated that all items constantly measure the same construct. Furthermore, contemporary research guarantees adequate convergent validity prior to the analysis of data. The construct measurement’s ability to represent the construct is called convergent validity [44]. Positive intercorrelation is anticipated among all indicators comprising a similar construct, given that they all reflect the same construct. [43] proposed that each construct attain an average variance extraction (AVE) of at least 50% to ensure adequate convergent validity. Table 2 describes the variables that yield sufficient AVE values, such as Employability Skills of Students (0.507), Quality Education (0.500), Course Structure (0.609), Institutional policy (0.585), and Physical Aspects (0.500). Hence, the present investigation satisfies the criteria for convergent validity.

#### Discriminant validity

Discriminant validity guarantees that the constructs within comparable frameworks are distinct from one another [43]. To ensure adequate discriminant validity, AVE’s square root must exceed the latent variable’s correlation [59], which are shown in Table 3.

**Table 3:** Discriminant validity analysis using Fornell and Lacker criterion* (Source: Authors).

**Constructs**	**CS**	**ES**	**PA**	**Pol**	**QE**
**CS**	**0.780**				
**ES**	0.546	**0.712**			
**PA**	0.626	0.599	**0.707**		
**Pol**	0.523	0.529	0.531	**0.765**	
**QE**	0.684	0.62	0.69	0.519	**0.733**

* **Note**: The square root of AVE is denoted by the bold values on the diagonal, whereas the other values represent the correlations between constructs. To satisfy the discriminant validity criteria, the diagonal values must be greater than the off-diagonal values.

### Structural model

#### Direct effect

Determination of structural model coefficients was accomplished via regression equations. When analysing structural relationships, the variance inflation factor (VIF) is commonly employed to ascertain whether an error is attributable to collinearity [60]. There are differing opinions among researchers regarding the optimal VIF threshold value. While [43] suggest a threshold of 5.0 [51], argue that a lower value, no more than 3, is preferable. The VIF value for each construct was equal to or less than three, indicating the absence of collinearity issues. The statistical significance of path coefficients was assessed through the bootstrapping method, with a minimal resampling of 5,000 [43]. The correlations between exogenous and endogenous components were computed using a significance level 0.05 (p<0.05). Thus, these results are shown in Table 4.

**Table 4:** Result of direct effect (path coefficient and hypothesis testing) (Source: Authors).

H	Path	Std. Beta	t-value	p-value	Decision	VIF	F2
**H1**	CS -> QE	0.057	0.647	0.518	Not Supported	2.427	0.004
**H2**	Pol -> QE	0.092	1.195	0.232	Not Supported	1.538	0.016
**H3**	PA -> QE	0.389	4.736	0	Supported	2.075	0.208
**H4**	QE -> ES	0.612	9.557	0	Supported	1	0.597

The direct effect of the relationship between CS and QE has not been supported in H1 (β = 0.057, T Value = 0.647, P Value = 0.518, F2 = 0.004, VIF = 2.427). The relationship between PA and QE is supported in H3 (β = 0.389, T Value = 4.736, P Value = 0.000, F2 = 0.208, VIF = 2.075). The relationship between Pol and QE is not supported in H2 (β = 0.092, T Value = 1.195, P Value = 0.232, F2 = 0.016, VIF = 1.538). The study of the relationship between QE and ES is supported in H4 (β = 0.612, T Value = 9.557, P Value = 0.000, F2 = 0.597, VIF = 1.000).

#### Testing the mediating effect

An investigation was undertaken to determine whether QE mediated the relationships between PA, CS, Pol and ES among students in higher education.

QE (H6: β = -0.238, t = 4.289, p = 0.000, H9: β = 0.235, t = 3.716, p = 0.000) mediated the relationship between PA, ES and ES in higher education. QE (H6, H7) did not mediate the relationship ES between CS and Pol. Thus, these results are shown in Table 5.

**Table 5:** The result of the mediating effect (Source: Authors).

Hypothesis	Path	Std. Beta	*t*-value	*p*-value	Decision
**H5**	PA -> QE -> ES	0.238	4.289	0.000	Supported
**H6**	CS -> QE -> ES	0.035	0.624	0.532	Not Supported
**H7**	Pol -> QE -> ES	0.056	1.167	0.243	Not Supported

#### Predictive relevance analysis (R2 & Q2)

The R2 value indicates the accuracy with which the independent variables of a model predict the dependent variable [61]. The R2 cut values of 0.75, 0.50, and 0.25 were categorised by [51] as significant, moderate, and feeble, respectively. The findings of this research demonstrate that the endogenous construct is explicable (QE: 0.650 and ES: 0.374), suggesting that the exogenous variables (PA, CS and Pol) account for around 37% and 65% of the variance, respectively. The suitability of this research model is certified by the R-square criterion.

Exogenous constructs are observed to predict endogenous constructs when the Q2 value is more significant than zero [44]. QE: 0.609 and ES: 0.415 indicate that the model is statistically highly predictive. The results presented in Table 6 demonstrate that the model possesses predictive validity.

**Table 6:** Predictive relevance of the exogenous constructs (Source: Authors).

Construct	R-square	Q-square
**ES**	0.374	0.415
**QE**	0.650	0.609

## Discussion

### Direct effects of constructs on quality education

This research contributes to the vital understanding of HEIs in Bangladesh and the student perception of how administrators and policymakers shape the curricula and other features. Prior literature has described and measured a solid direct effect of Course Structure, Physical Aspects, Institutional Policy, Quality Education and Employability Skills of Students. The current study has underlined similar findings as per the survey conducted on 154 students. Physical Aspects (PA) and Quality Education (QE) have all supported a direct effect with Quality Education (QE) and Employability Skills of Students (ES). [18] opined that the ‘tangibles’ (physical aspects) of any service determined the direction of the gap between perceived and experienced service. Some other items to which students agreed were "My university offers the facilities of my choice." (44.9%), "My university offers various support services." (46.2%), and "My university campus is attractive." (48.7%), and strongly agreed "I feel safe within my university campus." (45.5%). Thus, the hypothesis (H3), "Student perception of physical aspects positively affects the employability skills of higher education students”, has been proven. [15] argued that as students from diverse settings are coming to receive education at HEIs, the administration should emphasise outcome-based teaching and learning to decrease the gap between exceptional and slacking students. This observation closely matches the findings of this study because, once again, the majority of respondents agreed to the items "My university encourages professionalism and helps me foster the necessary skills" (50%), “My university is reputable and has enough credibility among the academic consensus” (52.6%), “My university is preparing me for a career of my choice” (39.1%), “The present curriculum design of my courses is coherent and clear to me” (56.4%), “Evaluation and assessment at my Institution is rigorous” (51.3%), and “My university actively involves students in their activities” (48.1%). Thus, the hypothesis (H4), "Student perception of Quality education positively affects the employability skills of higher education students", has been proven.

In contrast, the study failed to support the direct effect of Course Structure (CS) and Institutional Policy (POL) on Quality Education (QE). However, it should be noted that the p-values of both CS and POL are above zero but positive; thus, the observed data does not provide strong evidence against the null hypothesis [62], which is why the correlation may be positive against CS and POL. The findings provided by [16] in the Indian context indicated that content (Course Structure) had no significant difference in importance compared to other constructs when measuring student perception. Most respondents in this study agreed to the questions “The courses that I have taken are set to meet industry requirements” (50.6%), “My courses help me to foster communication skills and teamwork” (50.6%), and “The knowledge I am gaining is flexible across industries” (57.1%). However, by measuring the student perception in the context of Bangladesh, CS does not directly affect QE. While several reasons can be attributed to this difference, a likely explanation may be that the students in Bangladeshi HEIs either did not understand the question or lacked the experience required to answer this question entirely. [63] also found positive correlations when measuring the student perception of course assessment (Course Structure) but also measured the apparent competence of the students to evaluate this, thus proving that other attributes had affected the findings. [7, 8] observed widespread unemployment, pointing directly towards the lack of industry-based curricula in most HEIs and the opposite of the relatively positive student perception of CS. Thus, the hypothesis (H1), "Student perception of course structure positively affects the employability skills of higher education students", has not been proven.

Moreover [17], established that measuring program characteristics and effectiveness of Institutional Policy was nuanced because it was essential to follow a ’quantity-over-quality’ approach, which was not possible for the scope of this paper to accomplish. Similarly, the respondents mostly agreed to the questions including “My university gives opportunities to students to work as assistants” (46.8%), “My university gives me training on real-world work experiences” (44.9%) and "My university provides me with the knowledge that will be effective in my work life" (43.6%). this is not reflected by the findings. Since the study was conducted within Bangladesh, POL did not directly affect QE. Most HEIs are not invested in giving the students practical experience in their respective fields, owing to the vast number of unemployed students in Bangladesh [7, 8]. In practical terms, HEI policies such as assistantships, as observed by [64], made more students confident and able to perform tasks properly after specific assistantship roles than before, opposing the study’s findings. Thus, the hypothesis (H2), "Student perception of institutional policy positively affects the employability skills of higher education students", has not been proven.

### Mediating effect of quality education

The research also aimed to determine whether students perceive Quality Education (QE) as a mediating factor for ensuring Employability Skills of Students (ES) necessary to enter the workforce. This is significant as no prior literature discusses the mediating role of QE despite numerous pieces of evidence backing these claims [39–41]. Moreover, only those constructs that could support a direct relationship with QE also supported the mediated effect of QE. The current study underlines a significant finding surrounding the mediating effect of QE, as per the survey conducted on 154 students.

Physical Aspects (PA) is significantly supportive of the mediating effect of quality education for ensuring Employability Skills of Students (ES). While no prior literature within the scope of this study has argued or proven any significant relationship between PA and its effects on ES, HEI facilities have been extensively researched as a direct contributing factor to ensuring the ES of students [65–67]. This was mainly observed in the answers of respondents, as they overwhelmingly agreed that “My university offers the facilities of my choice” (44.9%) and “My university offers various support services” (46.2%) [67]. Even opined that "the availability of educational facilities determines how productive a lecturer will be in transforming the students into a qualified output that will continue with the national development of the country and nation at large". Thus, the hypothesis (H5) “Student perception of Quality education and the mediating relationship between physical aspects and employability skills of higher education students” has been proven.

Unfortunately, the findings do not support the mediating relationship of Quality Education (QE) to ensure Employability Skills (ES) for Course Structure (CS) and Institutional Policy (POL). This is unsurprising as CS and POL failed to support a direct effect relationship with ES. As mentioned, contextual differences and the students’ experience may have influenced the conflicting answers to the practical situation. Most respondents were optimistic that QE might mediate CS, as they agreed to the items "The courses that I have taken are set to meet industry requirements" (50.6%), "My courses help me to foster communication skills and teamwork" (50.6%), and "The knowledge I am gaining is flexible across industries" (57.1%). [68] corroborated similar findings as a vital link was established between course content (Course Structure) and vocational requirements (Employability Skills) since students were motivated by the vocational nature and saw future success in the employment market, however, was doubtful of its benefit in assessing the practical relationship between them. Thus, the hypothesis (H6), "Student perception of Quality education and the mediating relationship between course structure and employability skills of higher education students", has not been proven.

Moreover, respondents were also optimistic that QE would mediate POL by agreeing to the items "My university gives opportunities to students to work as assistants" (46.8%), "My university gives me training on real-world work experiences" (44.9%) and "My university provides me with knowledge that will be effective in my work life" (43.6%). [69] reiterated positive findings when implementing revised policies for HEIs in the European context, thus suggesting that the context of Bangladesh cannot connect with unrealistic student perceptions. Thus, the hypothesis (H7), "Student perception of Quality education and the mediating relationship between institutional policy and employability skills", has not been proven.

## Limitations of the study

While the paper forwards a significant contribution towards understanding the quality of education of private university students in Bangladesh, certain limitations still need to be addressed. This study used a cross-sectional approach. A longitudinal approach, wherein the competence of respondents (i.e. students) is measured before, though, and after the academic program, could provide us with a more holistic understanding of the subject matter. Secondly, the language of the questionnaire needed to be bilingual. However, it is safe to assume that private university students in Bangladesh are well-versed in English. However, as citizens of a country where English is not the native language, a bilingual questionnaire might have allowed the respondents to understand the items better. Thirdly, the study focused on respondents from only one private university, which could have limited the generalizability of the findings. Future researchers could focus on a broader sampling frame to enhance generalizability. Finally, publication bias may have been an issue in the present study, as reports with irrelevant conclusions may have yet to be disseminated.

## Conclusion

This research measured the quality of education within private universities in Bangladesh to ensure employability skills. Through a quantitative study of student perception, the paper further proposes a mediating role of quality education in shaping and ensuring the employability skills of HE students in Bangladesh. The study further underscores the significance of a holistic educational experience in fostering the diverse skill set demanded by the contemporary job market. Quality education, as perceived by students, extends beyond the traditional academic curriculum, emphasising the physical aspects, and policy of the university administration. These elements collectively contribute to the employability skills development of students and enhance their readiness for the workforce.

The findings reveal an alarming gap within Bangladesh’s education system that may even threaten the country’s economic growth and stability. As more students enrol in private HEIs, it must be impervious to the administration and faculty to ensure that a critical level of importance is given to industry-related skills within the courses taught and the facilities provided by the university. Students are key stakeholders in understanding these disparities. While it is understandable that different HEIs will have multiple challenges, the governing body for HEIs, the University Grants Commission (UGC), can implement strict policies to instil work-based learning and SCL to influence the development of employability skills in students.

The insights gained from this research can serve as a foundation for further investigations and initiatives to optimise the quality of education and enhance the employability skills of HE students in Bangladesh. Students must be made aware of the industry skills that they may learn from the courses offered by HEIs, which is only possible through the collective efforts of other stakeholders, culminating in industry-academia collaborations, curriculum enhancements, and a HE policy that maintains strict adherence to contemporary needs of the job market. The findings imply that HEIs in Bangladesh could focus on enhancing the quality of education by improving course structure, institutional policy, and physical aspects that could lead to higher employability skills among the graduates. This should assist HEIs in developing industry-ready global graduates.

As Bangladesh continues to navigate the challenges and opportunities presented by a rapidly evolving global economy, understanding the pivotal role of education in shaping a future-ready workforce becomes paramount. The role of HEIs is nurturing competitive candidates in the job industry.

## Supporting information

S1 Data (XLSX)
